# Thermodynamic analysis of thermal convection based on entropy production

**DOI:** 10.1038/s41598-019-46921-2

**Published:** 2019-07-17

**Authors:** Takahiko Ban, Keigo Shigeta

**Affiliations:** Division of Chemical Engineering, Department of Materials Engineering Science, Graduate School of Engineering Science, Osaka University, Machikaneyamacho 1-3, Toyonaka City, Osaka 560-8531 Japan

**Keywords:** Chemical engineering, Fluid dynamics

## Abstract

Flow patterns have a tendency to break the symmetry of an initial state of a system and form another spatiotemporal pattern when the system is driven far from equilibrium by temperature difference. For an annular channel, the axially symmetric flow becomes unstable beyond a given temperature difference threshold imposed in the system, leading to rotational oscillating waves. Many researchers have investigated this transition via linear stability analysis using the fundamental conservation equations and the generic model amplitude equation, i.e., the complex Ginzburg-Landau equation. Here, we present a quantitative study conducted of the thermal convection transition using thermodynamic analysis based on the maximum entropy production principle. Our analysis results reveal that the fluid system under nonequilibrium maximizes the entropy production induced by the thermodynamic flux in a direction perpendicular to the temperature difference. Further, we show that the thermodynamic flux as well as the entropy production can uniquely specify the thermodynamic states of the entire fluid system and propose an entropy production selection rule that can be used to specify the thermodynamic state of a nonequilibrium system.

## Introduction

Evaluating the stability of nonequilibrium states and finding a transition point between two nonequilibrium states require the construction of governing equations and subsequent detailed and laborious analysis of the equations^1–4^. Variational principles are quite significant for the analysis of phase behavior under nonequilibrium processes because of their broad application and the ease with which they are handled. The results of theoretical and experimental studies have suggested that nonequilibrium states maximize the entropy production of nonequilibrium processes (the so-called maximum entropy production principle (MEPP))^5–11^. The variational principles obtained from the MEPP^5^ yield well-established equations and various constitutive equations, such as the diffusion equation, Leslie–Ericksen equation, Nernst–Fokker–Planck equation, and constitutive equations describing complex phenomena, such as thin film hydrodynamics, colloid particles, kinetics of phase separation, deformation and diffusion in gels, nonlinear viscoelasticity of polymers, liquid crystals, and drying droplets^12–16^.

MEPP-based analysis has been extensively discussed for prediction of a transition point between two nonequilibrium states in complex systems; for example, those found in the configurational changes of crystal growth and the mode changes in droplet oscillation, which involves the two nonequilibrium processes interfering with each other, i.e., mass transfer and heat conduction, and mass transfer and viscous dissipation, respectively^6,7,11^. Nonequilibrium processes can be divided into two types: compound and complex^10^. For compound processes, the uncoupled processes are decomposed into various elementary processes that are only dependent on the corresponding thermodynamic flux, and not on all of the fluxes. All of the entropy production in compound processes can be represented as the sum of the functions of all of the elementary processes. For complex processes, various elementary processes are coupled and interfere with each other. Previous studies that attempted to disprove the MEPP are outside the range of MEPP applicability because the nonequilibrium systems investigated are compound systems^17–22^. It is claimed that the MEPP is valid for complex systems and invalid for compound systems^23^. The MEPP indicates that the systems evolve such as to maximize their entropy production. However, it is unclear whether partial or total entropy production is an essential factor to determine the time course of the systems. Further, previous studies have not explained why a certain component of entropy production can provide a selection rule that determines the nonequilibrium states although different entropy production components originate from various irreversible processes occurring within a nonequilibrium system. The recent literatures on convection experiments demonstrate that the realized convection patterns are not governed by principles like the MEPP^24–26^. Thus, it was stated that the MEPP has been applied in a largely ad hoc manner and its successes remain something of an unexplained curiosity^27^.

In this study, we evaluated the relationship between the flow patterns and entropy production via numerical simulation to develop an entropy production selection rule that can be used to specify the thermodynamic state of a nonequilibrium system. We consider the flow patterns driven by thermocapillary instabilities as a complex process involving two irreversible processes: viscous dissipation and heat conduction. The temperature difference imposed across the cell induces a surface tension gradient on the free surface of the fluid, leading to a surface flow towards the cold side. The resulting thermal convection induces a various flow patterns with an internal circulation. In the case of an annular channel, the flow pattern is axially symmetric along the temperature gradient with an internal circulation. This axially symmetric flow (ASF) becomes unstable beyond a given temperature difference threshold and subsequently symmetry-breaking flow, i.e., rotational oscillating waves, appears. The oscillating waves propagate in a direction perpendicular to the temperature gradient applied to the system, i.e., in the circumferential direction, and the temperature changes periodically. This rotational oscillating flow is called hydrothermal wave (HTW)^28–30^. The research of the transition from ASF to HTW has been stimulated by numerical experiments on the fundamental conservation equations, i.e., the Navier-Stokes equation and conservations of mass and energy, and the generic model amplitude equation, i.e., the complex Ginzburg-Landsu equation. Remarkable predictions concern the existence of various flow patterns and instabilities^31,32^. In this study, we first computed each component of thermodynamic flux and thermodynamic force in both ASF and HTW to calculate each component of entropy production. Then, we ascertained which component of thermodynamic flux can specify the system, and which component of entropy production can be maximized to predict the system behavior.

We assume that a linear relationship between the thermodynamic force and the thermodynamic flux holds for the two irreversible processes considered in this study. Then, for heat conduction, the thermodynamic force is **X**_T_ = **∇**(1/*T*), and the thermodynamic flux is **J**_T_ = −*λ***∇***T*, where *λ* is the thermal conductivity and *T* is the temperature; for viscous dissipation of the fluid, they are **X**_V,*ij*_ = *σ*_*ij*_/*T* and **J**_V,*ij*_ = ∂*u*_*i*_/∂*x*_*j*_, respectively, where *σ*_*ij*_ is the viscous stress tensor, *u*_*i*_ is the *i-th* component of the fluid velocity vector ***u***, and *x*_*j*_ is the *j-th* component of coordinates. The entropy production of each elementary process can then be calculated as the product of the thermodynamic force and thermodynamic flux. We find that the ratio of both entropy productions, i.e., for heat conduction (*σ*_T_) and viscous dissipation (*σ*_V_), is *σ*_V_/*σ*_T_ ≈ 10^−7^. The energy dissipation due to the viscosity is much less than the entropy production due to heat conduction; therefore, we ignore the energy dissipation in this study. Note that this question of ignoring the smaller entropy production requires careful consideration and will be discussed in a later work. Further, in the analysis presented below, we omit the subscripts *T* and *V* from the physical quantities because we focus on the entropy production due to heat conduction only.

We take the time-averaged values of the local thermodynamic flux during a certain period *τ* as $|\,{J}_{i}|=1/\tau {\int }_{{t}_{o}}^{{t}_{o}+\tau }\sqrt{{(\lambda {\rm{\nabla }}T)}^{2}}dt,$ and the local thermodynamic force as $|{X}_{i}|=1/\tau {\int }_{{t}_{o}}^{{t}_{o}+\tau }\sqrt{{(\nabla (1/T))}^{2}}dt,$ where *i* = *x*, *y*, *z*, because the thermodynamic variables vary spatiotemporally in the nonequilibrium system. Most variables are measured at the point (*x*, *y*, *z*) = (30 mm, 0 mm, 3 mm). The *x* component of the heat flux is in the same direction as Δ*T* between the inner and outer walls. We calculate the absolute value of the local entropy production from the following relation,

1 $${\sigma }_{i}=1/\tau {\int }_{{t}_{o}}^{{t}_{o}+\tau }|{J}_{i}||{X}_{i}|dt,$$

where *J*_*i*_*X*_*i*_ denotes the simple product of *J*_*i*_ and *X*_*i*_. Note that the entropy production is scalar, and *σ*_*i*_ represents the entropy production generated by the *i*th component of the heat flux. The total entropy production is the sum of the entropy production contributions for all components:

$${\sigma }_{{\rm{total}}}=\sum _{i}{\sigma }_{i}.$$

**Figure 1:**
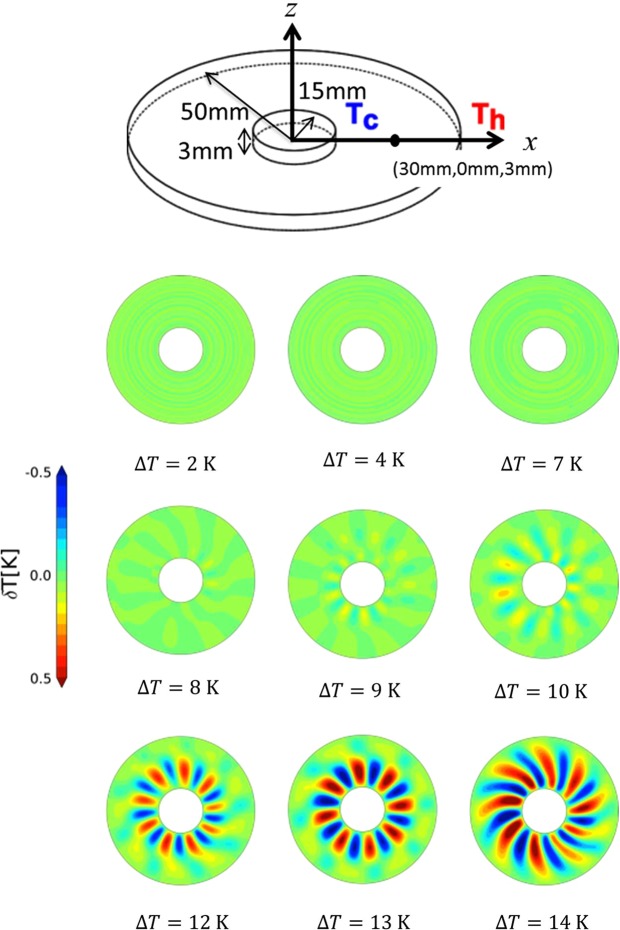
Schematics of the annular container (upper panel). Surface temperature difference at various temperature differences and at *t* = 150 s (lower panels).

Figure 1 shows the computed surface temperature fluctuation, *δT*, in the horizontal plane, which is defined by $\delta T({\bf{x}},\,t)=T({\bf{x}},\,t)-1/2\pi {\int }_{0}^{2\pi }T({\bf{x}},\,t)d\theta .$ When the temperature difference Δ*T* is less than 7 K, an axially symmetric flow (ASF) occurs. However, as Δ*T* increases above 8 K, traveling waves propagate in the direction perpendicular to the temperature gradient applied to the system, i.e., in the circumferential direction, and the temperature changes periodically. This rotational oscillating flow is called an hydrothermal waves (HTW)^28–30^. In a temperature range for which an HTW is produced, the surface temperature started to vibrate at about 50–100 s after the start of the simulation. The amplitude of the temperature oscillation approached a constant value over time, and, after 400 s, the wave number of the transmission wave also became a constant value with the standard deviation of the amplitude being less than 0.01 K. However, even if the simulation was performed for 2500 s, the amplitude of the temperature oscillation did not become constant, and there was a standard deviation of 0.0008 K. Considering the calculation cost, we analyzed the two intervals of 200–300 s and 300–400 s in this study.

## Results and Discussion

**Figure 2:**
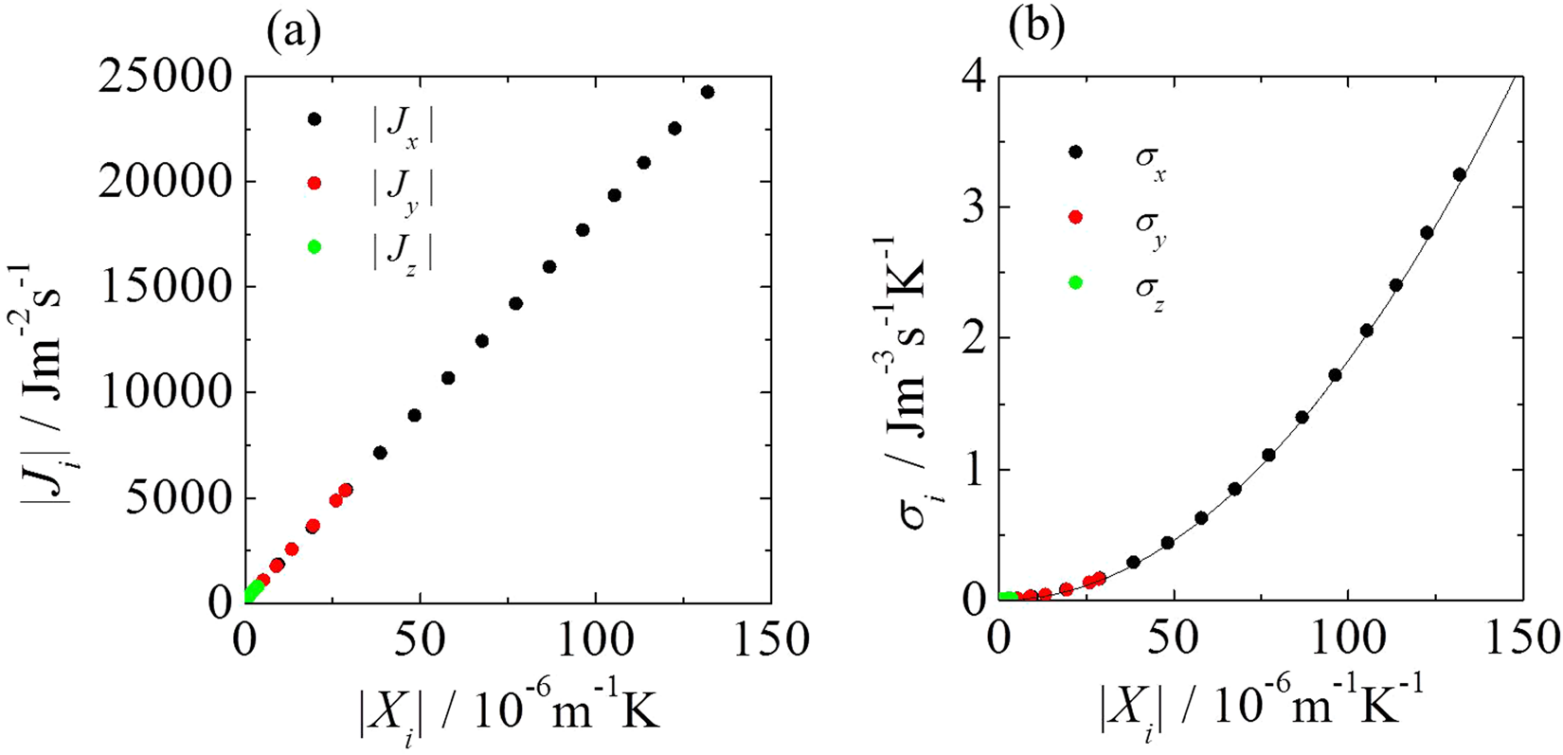
(**a**) Relationship between the local thermodynamic flux *J*_*i*_ and the local thermodynamic force *X*_*i*_. (**b**) Relationship between the entropy production *σ*_*i*_ and the local thermodynamic force *X*_*i*_ for an ASF and HTW. The solid line is fit using the quadratic curve, ${\sigma }_{i}=1.83\times {10}^{8}{{X}_{i}}^{2}$. It is in agreement with the theoretical curve, ${\sigma }_{i}={\rm{\lambda }}{T}^{2}{{X}_{i}}^{2}$.

First, we investigated the relationship between |*J*_*i*_| and |*X*_*i*_| (Fig. 2a). The relationship is a single line through the origin, although the flow pattern changes from ASF to HTW. Moreover, the relationship between each entropy production component *σ*_*i*_and |*X*_*i*_| is a single quadratic curve, ${\sigma }_{i}=1.83\times {10}^{8}{{X}_{i}}^{2}$ (Fig. 2b). If the entropy production can be described by the Fourier law as due to heat conduction only, it can be expressed as ${\sigma }_{i}={\rm{\lambda }}{T}^{2}{{X}_{i}}^{2}$. Hence, we obtain *σ*_*i*_ = 1.82 − $1.84\times {10}^{8}{{X}_{i}}^{2}$ (we respectively used the minimum and maximum values of temperature for calculation because the temperature oscillates). This relation is consistent with the fitting curve in Fig. 2b. The results indicating that the single curves describe these relationships are justified by the fact that the thermodynamic flux is defined on the basis of a linear function of the thermodynamic force. Thus, for the entropy production expressed as a function of *X*_*i*_, the MEPP is inapplicable for specifying the thermodynamic states and for predicting the transition point from an ASF to an HTW.

We must therefore query how the MEPP successfully predicted the transition points of the nonequilibrium states in the experiments reported in refs^11,16,17^. In those experiments, instead of a local thermodynamic force, a driving force adjusted by the energy supplied from the surroundings was used as a thermodynamic force; for example, the degree of supersaturation, the degree of supercooling, and a pressure gradient were used. The driving force, which is constant in time, serves to prevent the internal system state from achieving thermal equilibrium. As the driving force increases, the system state changes. Thus, the driving force serves as a measure of the nonequilibrium degree. For our system, we next analyze the system’s behavior using the thermodynamic variables as functions of the driving force, which is determined by the temperature gradient between the inner and outer walls, i.e., *F* = (1/*T*_c_ − 1/*T*_h_)/(*R*_o_ − *R*_i_). Note that *F* has the same dimension as a thermodynamic force owing to heat conduction.

**Figure 3:**
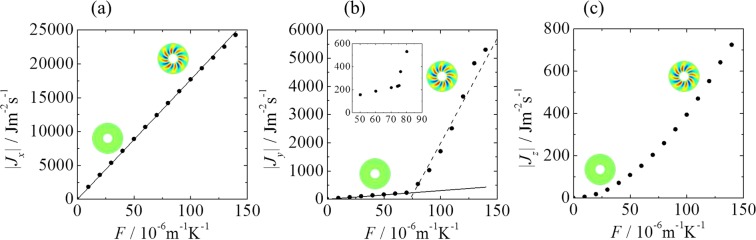
Effect of the driving force *F* on each component of the local thermodynamic flux *J*_*i*_ for an ASF and HTW: (**a**–**c**) *J*_*x*_, *J*_*y*_, and *J*_*z*_, respectively. (**a**) The solid line represents *J*_*x*_ = λ*T*_c_*T*_h_(*R*_o_ − *R*_i_)*F*/*r*ln(*R*_o_/*R*_i_). (**b**) The solid and broken lines are two regression lines calculated from the relation *σ*_*y*_ = *J*_*y*_*X*_*y*_ (See the text for more details). Inset: Magnified view.

Figure 3 shows the relationships between the driving force and each component of the local thermodynamic flux. The flow pattern changes from an ASF to an HTW with a jump in |*J*_*y*_| at a driving force of *F* = 70–80 × 10^−6^ m^−1^K^−1^, corresponding to Δ*T* ≈ 7–8 K. Quantitative analysis reveals that for an ASF, the first derivative of |*J*_*y*_| remains constant at the value of (3.07 ± 0.00(4)) × 10^6^ Jm^−1^s^−1^K^−1^, whereas for an HTW, the value is (74.0 ± 27.3) × 10^6^ Jm^−1^s^−1^K^−1^. In each flow pattern, |*J*_*y*_| can be expressed by two straight lines with significantly different slopes as functions of *F*. For *J*_*x*_, regardless of whether the flow pattern is an ASF or HTW, this term increases monotonically with respect to *F*. The values can be expressed using the theoretical relation *J*_*x*_ = λ*T*_c_*T*_h_(*R*_o_ − *R*_i_)*F*/*R*ln(*R*_o_/*R*_i_), where *R*(=30mm) is the measurement point. Finally, *J*_*z*_ slightly changes its derivatives, although it is not as well determined as *J*_*y*_. From a quantitative analysis conducted, we found that for *J*_*z*_, the slope of its derivative of an ASF region decreases by 41.8% in comparison with that of an HTW region. Interestingly, *J*_*y*_ and *J*_*z*_ are in a direction perpendicular to *F* and they exhibit a nontrivial change with *F*. HTW occurs owing to the symmetry-breaking of heat conduction. The thermodynamic fluxes perpendicular to *F* may provide a clue to a signature of the change in the flow patterns. According to the MEPP, a jump in the thermodynamic flux is the signature of a first order transition^33^. Therefore, we can distinguish one nonequilibrium state from another in terms of the thermodynamic fluxes with respect to the driving force. As *J*_*y*_ can be expressed by the linear function of *F*, the absolute value of entropy production calculated from Eq. (1) can be fitted by the following quadratic function:

2 $${\sigma }_{i}(F)={L}_{i}{(F-{\theta }_{i})}^{2},$$

where *L*_*i*_ and *θ*_*i*_ are phenomenological coefficients^6,11,33^. The entropy production in each state can be expressed by different quadratic curves, i.e., for an ASF and HTW. We can also fit the experimental values of *J*_*y*_ using ${J}_{y}(F)=\sqrt{L{L}_{y}}(F-{\theta }_{y}),$ calculated from the relation ${\sigma }_{y}={J}_{y}{X}_{y}={{J}_{y}}^{2}/L,(L=\lambda {T}^{2})$.

**Figure 4:**
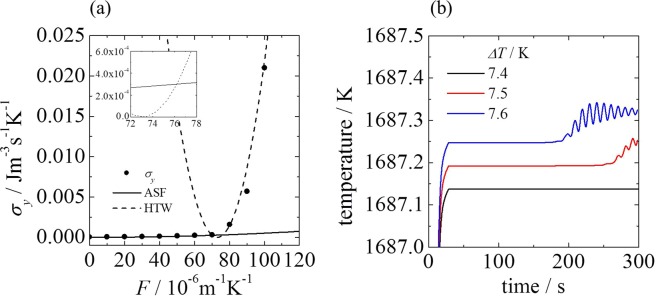
(**a**) Effect of the driving force *F* on the *y*-component of the entropy production *σ*_*y*_ for an ASF and HTW. The solid and broken curves represent the regression curves for an ASF and HTW using Eq. (2): *y* = 5.18 × 10^−8^*x*^2^, and *y* = 3.00 × 10^−5^(*x* − 73.0)^2^, respectively. The intersection point of the two curves for the ASF and HTW correspond to the transition point. Inset: Magnified view. (**b**) Time course of the temperature *T* near the prediction points. Δ*T* is the temperature difference between the inner and outer walls.

The relationship between *σ*_*y*_ and *F* is shown in Fig. 4a. In the low-thermodynamic-force region, the ASF entropy production curve lies above that of the HTW, whereas the opposite is true for the high-thermodynamic-force region. The intersection point of the two curves is at *F*_*c*_ = 76.2 × 10^−6^ m^−1^K^−1^, which corresponds to Δ*T*_*c*_ = 7.59K when the fitting range for the HTW entropy production is 8–11 K (Δ*T*_*c*_ = 8.15 K for the 8-14-K range). We can interpret the system behavior according to the MEPP. That is, the ASF appeared below the point of intersection because the entropy production for the ASF exceeded that for the HTW. Above the point of intersection, the entropy production for the HTW became greater. The system changed to maximize the entropy production induced by *J*_*y*_ with respect to *F*. Thus, the transition point is the intersection point of the two curves for the ASF and HTW. Note that all absolute values of *σ*_*y*_ described here are the same values as *σ*_*y*_ described in Fig. 2(b). The only difference between the two entropy productions is the variable expressed by the function of *X* or *F*. *σ*_*y*_ with respect to *F* can be described as the two different curves in each flow pattern, whereas *σ*_*y*_ with respect to *X* falls on a single curve. The function *σ*_*y*_(*F*) permits the distinction between the two flow patterns. *θ*_*i*_ in Eq. (2) is an important factor because, in the case of a nonzero value, state transition occurs. The physical meaning of *θ*_*i*_ was interpreted as a correction term for conversion to the local driving force^6^. Our results shown in Fig. 3(a) rule out this interpretation. Thus, *θ*_*i*_ may describe the interference of the two irreversible processes or the degree of symmetry-breaking because for *σ*_*x*_, *θ*_*x*_ = 0. Full interpretation of the physical meaning needs to be investigated further.

To verify the transition point determined by the MEPP, we computed the time course of the temperature at the measurement position by varying Δ*T* in intervals of 0.1 K (Fig. 4b). At Δ*T* = 7.4 K, the temperature remained constant during the measurement time span. At Δ*T* = 7.5 K, however, the temperature started to increase at a time of approximately 260 s. Eventually, temperature oscillation began. This result indicates that HTW behavior appeared at Δ*T* = 7.5 K, and this transition point is extremely close to the value calculated from the MEPP. Although the calculation using the MEPP was performed in 1-K intervals, the precision of the predicted value is extremely high (note that we obtain complete agreement on the transition point if we set the intervals to 0.5 K). The MEPP correctly predict the transition point only in the time span in which the MEPP analysis is conducted. In fact, for Δ*T* = 7.4 K, the temperature oscillated at a time of approximately 400 s, and the HTW appeared. When the analysis period was changed from 200–300 s to 300–400 s, we found that the prediction point calculated by the MEPP decreased by approximately 0.1 K. These results indicate that, although the nonequilibrium system does not reach a steady state, the MEPP can specify the nonequilibrium state and predict the transition point.

**Figure 5:**
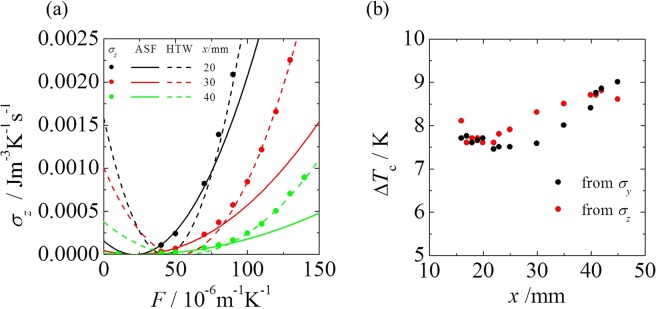
(**a**) Entropy production for the *z* components, *σ*_*z*_, as functions of the driving force *F* at various measurement points. The solid and broken lines represent the fitting curves of the entropy production for an ASF and HTW, respectively, obtained using Eq. (2). The intersection point of the two curves for the ASF and HTW correspond to the transition point. (**b**) Comparison of the transition-point prediction based on analysis of *σ*_*y*_ and *σ*_*z*_ at various measurement points.

This understanding motivates the question of how *J*_*y*_ is capable of uniquely specifying the nonequilibrium states. The decisive component of the thermodynamic variables that specifies the nonequilibrium states may be the component that breaks the spatial symmetry with respect to the driving force applied to the entire system. *J*_*y*_ at the measurement point is perpendicular to the driving force. In context, using *J*_*z*_, we must be able to predict the transition point because *J*_*z*_ is perpendicular to the driving force. Therefore, using the same approach as that for the calculation of *σ*_*y*_, we analyzed the transition points obtained from the *σ*_*z*_ component at different measurement points. For simplicity, we fit *σ*_*z*_ using a quadratic function of *F*, even though *J*_*z*_ may behave as a high-order dependency of *F*. The result is shown in Fig. 5.*σ*_*z*_ exhibits the distinctive features exhibited by *σ*_*y*_: first, the system changes to maximize the entropy production; and second, the point of state transitions coincides with the intersection between the entropy production curves. These transition points are congruent with those obtained from the *σ*_*y*_ component (Fig. 5b).

**Figure 6:**
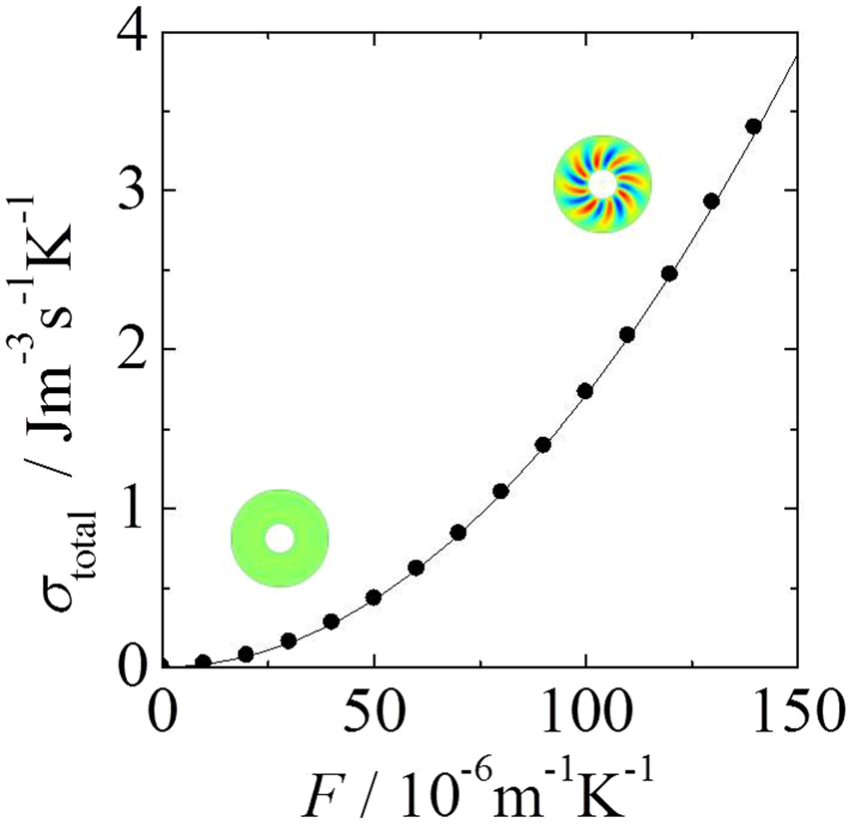
Relationship between the total entropy production and the driving force *F*. The solid curve represents *σ*_*x*_ = *λT*_*c*_*T*_*h*_[(*R*_*o*_ − *R*_*i*_)*F*/*R*ln(*R*_*o*_/*R*_*i*_)]^2^.

We investigated the relationship between the driving force *F* and the total entropy production (Fig. 6). The total entropy production falls on a single curve, ${\sigma }_{x}={\rm{\lambda }}{T}_{{\rm{c}}}{T}_{{\rm{h}}}{[({R}_{{\rm{o}}}-{R}_{{\rm{i}}})F/R\mathrm{ln}({R}_{{\rm{o}}}/{R}_{{\rm{i}}})]}^{2}$, which represents the theoretical value of the *x* component of entropy production expressed as a function of *F*. We unsuccessfully attempted to predict the transition point using the total entropy production as a function of *F* because the total entropy production increases monotonically with *F*. We found that information related to the system was lost because the *σ*_*x*_ component, which constitutes 94.6–99.9% of the total entropy production, covers the slight change in the entropy production due to the transition. This finding indicates that the largest component of the entropy production as well as the total entropy production cannot distinguish one nonequilibrium state from another. The results indicate that the current formulation of the MEPP requires revision.

From the above results, we deduced that a specific component of the thermodynamic flux depending on the driving force distinguishes one nonequilibrium state from another. Hence, we may extend the MEPP using the relation between the thermodynamic flux and the driving force. This relation specifies the nonequilibrium states as thermodynamic phases similar to phases in equilibrium. Therefore, we can derive an equation related to the phase boundaries between the nonequilibrium states. Using the corrected thermodynamic force *I*_*i*_ = (*F*_*i*_ − *θ*_*i*_), instead of the local thermodynamic force^6,11,33^ and its conjugate variable *J*_*i*_(*F*_*i*_), the maximum entropy production principle can be rewritten as

3 $${\rm{\delta }}[\sigma ({F}_{k})-\mu (\sigma ({F}_{k})-\sum _{i}{I}_{i}{J}_{i})]=0,$$

where *μ* is a Lagrangian multiplier. We obtain the following explicit expression for the thermodynamic flux:

4 $${J}_{i}({F}_{i})=\frac{\sigma }{{\sum }_{i}{I}_{i}(\partial \sigma /\partial {F}_{i})}\frac{\partial \sigma }{\partial {F}_{i}}=\frac{1}{{r}_{i}}\frac{\partial \sigma }{\partial {F}_{i}}.$$

Coefficient *r*_*i*_ corresponds to the degree of a homogeneous function^5^. If two different driving forces, *F*_1_, *F*_2_, are applied to a system, the entropy production can be expressed in the form *σ* = *σ*(*F*_1_, *F*_2_). Then, the differential form of the entropy production can be given as

5 $$d\sigma =(\frac{\partial \sigma }{\partial {F}_{1}})d{F}_{1}+(\frac{\partial \sigma }{\partial F})d{F}_{2}.$$

This equation holds for different nonequilibrium phases. When two nonequilibrium phases are at a transition point, the entropy productions of the two phases are equal. Therefore, from Eqs (4) and (5), we obtain

6 $$\frac{d{F}_{1}}{d{F}_{2}}=-\,\frac{{r}_{2}\Delta {J}_{2}({F}_{2})}{{r}_{1}\Delta {J}_{1}({F}_{1})},$$

where Δ*J* is the difference in the thermodynamic fluxes for the transition from one phase to another. This expression may correspond to the Clausius–Clapeyron equation describing the slope of the phase boundaries in equilibrium.

In this paper, the relationship between the thermodynamic flux and the various nonequilibrium states in a system incorporating two irreversible processes were examined. A nonequilibrium state changes to another state to maximize the entropy production induced by the thermodynamic flux in a direction perpendicular to the driving force. Hence, a selection rule for predicting the transition point between different states is needed to evaluate the symmetry-breaking component of the thermodynamic flux with respect to the driving force. Moreover, our analysis revealed that the total entropy production and the largest entropy production cover the slight change in the entropy production due to the transition. Thus, the quantities involved in this principle are likely to be system-dependent.

One wonders if the MEPP-based analysis can apply to nonequilibrium systems involving several branches of solutions, for example, bistability in flow patterns^3,34^. A slight difference in initial perturbation leads to a completely different patterns. In this manuscript, we used the steady value of driving force applied to the entire system as a kind of state variable describing entropy production and thermodynamic flux. This approach cannot be used for nonequilibrium systems exhibiting several branches of solutions because the same value of driving force may correspond to different entropy productions exhibited by different branches. We may use driving force reflecting initial conditions as state variables to predict the system behavior; the difference in the initial perturbation corresponds to the difference in the driving force. Therefore, when we use an initial value of driving force or its average value as nonlocal thermodynamic force, different values of the nonlocal thermodynamic force may specify different branches of solutions. If all branches of solutions show the same entropy production, the approach predicts that coexisting states will appear. If there is a slight difference in entropy production among several branches of solutions, the branch exhibiting the maximum entropy production will be selected. Consequently, applying our modified MEEP to nonequilibrium systems involving several branches of solutions is a significant issue to be addressed. Finally, we proposed an equation related to the phase boundaries between the nonequilibrium states on the basis of the MEPP involving entropy production as a function of the driving force. Experimental and numerical validation of this equation will reveal the range of MEPP applicability.

### Numerical calculation methods

We investigate the thermal convection in an annular pool filled with silicon melt (height: 3 mm), for which there is a fixed temperature difference between the inner (*R*_i_ = 15 mm) and outer walls (*R*_o_ = 50 mm). The silicon melt is a noncompressible Newtonian fluid. We used the continuity equation as the governing equation along with the Navier–Stokes equation and energy equation:

7 $$\nabla \cdot {\boldsymbol{u}}=0,$$

8 $$\frac{\partial {\boldsymbol{u}}}{\partial t}+{\boldsymbol{u}}\cdot \nabla {\boldsymbol{u}}=-\frac{1}{\rho }\nabla {\boldsymbol{p}}+\nu {\nabla }^{2}{\boldsymbol{u}},$$

9 $$\frac{\partial T}{\partial t}+{\boldsymbol{u}}\cdot \nabla T=\alpha {\nabla }^{2}T,$$

where ***u*** is the fluid velocity vector, *t* is the time, *ρ* is the density of the silicone melt, ***p*** is the pressure, *ν* is the kinetic viscosity of the silicone melt, *T* is the temperature, and *α* is the thermal diffusivity of the silicon melt. According to order analysis, the strength of natural convection relative to Marangoni convection can be determined by the parameter *Ra*^1/2^/*Ma*^2/3^(<1, *Ra*: Rayleighnumber, *Ma*: Marangoninumber). In this system, this value is small; therefore we ignored the effect of the gravity term in the Navier–Stokes equation. The boundary conditions at the free surface and at the container bottom are expressed by the following equations:

$$z=d:$$

$$\begin{array}{cccc}{u}_{z}=0, & \mu \frac{\partial {u}_{x}}{\partial z}=-\,{\sigma }_{T}\frac{\partial T}{\partial x}, & \mu \frac{\partial {u}_{y}}{\partial z}=-\,{\sigma }_{T}\frac{\partial T}{\partial y}, & \frac{\partial T}{\partial z}=0;\end{array}$$

$$z=0:$$

$$\begin{array}{cc}{u}_{x}={u}_{y}={u}_{z}=0, & \frac{\partial T}{\partial z}=0.\end{array}$$

For the boundary condition at the free surface, we considered the generation of convection due to the thermal Marangoni effect. We also assumed that the shape of the free surface would not change. Solid walls were used for the inner wall, outer wall, and bottom surface of the container, and we used a no-slip condition for the velocity. We fixed the temperature of the inner wall to be constant and set the outer wall to a temperature higher than that of the inner wall. Specifically, we set the temperature of the inner wall to *T*_*c*_ = *T*_*m*_ = 1683 K (*T*_*m*_: melting point of silicon), and we varied the temperature of the outer wall in the range of *T*_*h*_(=Δ*T* + *T*_c_) = 1684 K to 1697 K. We used adiabatic conditions for the upper surface and bottom surface. For the initial conditions, we set the velocity of the fluid within the container to 0, and the temperature was homogeneous at a value of 1683 K.

We set the number of grid points to be 81 in the radial direction, 180 in the circumferential direction, and 21 in the vertical direction. These numbers for the grid are based on the conditions used by Li *et al*.^35^, and the resolution was sufficient to handle hydrothermal waves for a fluid with a low Prandtl number. We discretized the equations with the finite volume method and performed the numerical calculations using OpenFOAM, which uses the PISO algorithm. The details of the calculation method have been described in previous research^36^. The simulation code has already been verified, and we verified that there were no problems. The physical values used in the calculations were the same as the values used previously, as presented in Tables 1 and 2.

**Table 1:** Physical properties.

Physical properties	Value
Thermal conductivity (*λ*)	64 Wm^−1^K^−1^
Viscosity (*μ*)	7.0 × 10^−4^ kgm^−1^s^−1^
Density (*ρ*)	2530 kgm^−3^
Thermal expansion coefficient (*β*)	1.5 × 10^−4^ K^−1^
Surface tension coefficient (*γ*_T_)	−7.0 × 10^−5^ Nm^−1^K^−1^
Heat capacity (*C*_p_)	1000 Jkg^−1^K^−1^
Melting temperature (*T*_m_)	1683 K

**Table 2:** Numerical parameters of the simulation.

Parameter	Value
Grashof number (*Gr* = *gρ*^2^*β*Δ*Td*^3^/*μ*^2^)	1.19 × 10^4^
Prandtl number (*Pr* = *μC*_*p*_/*λ*)	1.09 × 10^−2^
Marangoni number (*Ma* = −*γ*_*T*_Δ*T*(*R*_o_ − *R*_i_)/*μα*, *α* = *λ*/*ρC*_*p*_)	5.54 × 10^2^ − 2.08 × 10^3^
Rayleigh number (*Ra* = *Gr* × *Pr*)	1.19 × 10^2^

## References

[1] Chandrasekhar, S. *Hydrodynamic and Hydromagnetic Stability*. (Oxford University Press, 1961).

[2] Malkus WVR, Veronis G (1958). Finite amplitude cellular convection. J. Fluid Mech..

[3] Busse BFH (1967). The stability of finite amplitude cellular convection and its relation to an extremum principle. J. Fluid Mech..

[4] Graham R (1974). Hydrodynamic fiuctuations near the convection instability. Phys. Rev. A.

[5] Martyushev LM, Seleznev VD (2006). Maximum entropy production principle in physics. chemistry and biology. Phys. Rep..

[6] Martyushev LM (2007). Some interesting consequences of the maximum entropy production principle. J. Exp. Theor. Phys..

[7] Ban T, Hatada Y, Horie K (2014). Thermodynamic Study on the Mode Change in Droplet Oscillation Arising from the Marangoni Effect. Kagaku Kogaku Ronbunshu.

[8] Belkin A, Hubler A, Bezryadin A (2015). Self-assembled wiggling nano-structures and the principle of maximum entropy production. Sci. Rep..

[9] Ziegler, H. *Progress in Solid Mechanics*. *North Holland, Amsterdam* (North Holland, Amsterdam, 1963).

[10] Santonico R. (2016). An introduction to RPC2016. Journal of Instrumentation.

[11] Hill A (1990). Entropy production as the selection rule between different growth morphologies. Nature.

[12] Onsager L, Fuoss RM (1931). Irreversible Processes in Electrolytes. Diffusion, Conductance and Viscous Flow in Arbitrary Mixtures of Strong Electrolytes. J. Phys. Chem..

[13] Doi M (2011). Onsager’s variational principle in soft matter. J. Phys. Condens. Matter.

[14] Xu X, Thiele U, Qian T (2015). A Variational approach to thin film hydrodynamics of binary mixtures. J. Phys. Condens. Matter.

[15] Man X, Doi M (2016). Ring to Mountain Transition in Deposition Pattern of Drying Droplets. Phys. Rev. Lett..

[16] Man X, Doi M (2017). Vapor-Induced Motion of Liquid Droplets on an Inert Substrate. Phys. Rev. Lett..

[17] Andresen B, Zimmermann EC, Ross J (1984). Objections to a proposal on the rate of entropy production in systems far from equilibrium. J. Chem. Phys..

[18] Ross J, Corlan AD, Müller SC (2012). Proposed principles of maximum local entropy production. J. Phys. Chem. B.

[19] Vellela M, Qian H (2009). Stochastic dynamics and non-equilibrium thermodynamics of a bistable chemical system: the Schlogl model revisited. J. R. Soc. Interface.

[20] Nicolis C, Nicolis G (2010). Stability, complexity and the maximum dissipation conjecture. Q. J. R. Meteorol. Soc..

[21] Meysman FJR, Bruers S (2010). Ecosystem functioning and maximum entropy production: a quantitative test of hypotheses. Philos. Trans. R. Soc. B Biol. Sci..

[22] Polettini M (2013). Fact-checking Ziegler’s maximum entropy production principle beyond the linear regime and towards Steady States. Entropy.

[23] Martyushev LM, Seleznev VD (2014). The restrictions of the maximum entropy production principle. Phys. A Stat. Mech. its Appl..

[24] Egolf DA, Melnikov IV, Pesch W (2000). Mechanisms of extensive spatiotemporal chaos in Rayleigh–Bénard convection. Nature.

[25] Cakmur RV, Egolf DA, Plapp BB, Bodenschatz E (1997). Bistability and Competition of Spatiotemporal Chaotic and Fixed Point Attractors in Rayleigh-Bénard Convection. Phys. Rev. Lett..

[26] Daniels KE, Beck C, Bodenschatz E (2004). Defect turbulence and generalized statistical mechanics. Phys. D Nonlinear Phenom..

[27] Dewar Roderick C., Lineweaver Charles H., Niven Robert K., Regenauer-Lieb Klaus (2014). Beyond the Second Law.

[28] Mukolobwiez N, Chiffaudel A, Daviaud F (1998). Supercritical eckhaus instability for surface-tension-driven hydrothermal waves. Phys. Rev. Lett..

[29] Schwabe D, Möller U, Schneider J, Scharmann A (1992). Instabilities of shallow dynamic thermocapillary liquid layers. Phys. Fluids A.

[30] Smith MK (1986). Instability mechanisms in dynamic thermocapillary liquid layers. Phys. Fluids.

[31] Shraiman BI (1992). Spatiotemporal chaos in the one-dimensional complex Ginzburg-Landau equation. Phys. D Nonlinear Phenom..

[32] Hoyas S, Gil A, Fajardo P, Pérez-Quiles MJ (2013). Codimension-three bifurcations in a Bénard-Marangoni problem. Phys. Rev. E - Stat. Nonlinear, Soft Matter Phys..

[33] Martyushev LM, Konovalov MS (2011). Thermodynamic model of nonequilibrium phase transitions. Phys. Rev. E - Stat. Nonlinear, Soft Matter Phys..

[34] Meyer CW, Cannell DS, Ahlers G (1992). Hexagonal and roll flow patterns in temporally modulated Rayleigh-Benard convection. Phys. Rev. A.

[35] Li YR, Imaishi N, Azami T, Hibiya T (2004). Three-dimensional oscillatory flow in a thin annular pool of silicon melt. J. Cryst. Growth.

[36] Takagi Y, Okano Y, Minakuchi H, Dost S (2014). Combined effect of crucible rotation and magnetic field on hydrothermal wave. J. Cryst. Growth.

